# Evaluating the long-term efficacy of primary surgical repair in communicating Debakey IIIB chronic dissecting aortic aneurysms

**DOI:** 10.1016/j.xjon.2024.08.001

**Published:** 2024-08-19

**Authors:** FNU Venjhraj, Ajeet Singh, Ravi Das, Ashvin Kumar

**Affiliations:** a Department of Internal Medicine, Shaheed Mohtarma Benazir Bhutto Medical College Lyari, Karachi, Pakistan; b Department of Internal Medicine, Dow University of Health Sciences, Karachi, Pakistan; c Jinnah Sindh Medical University, Karachi, Pakistan

To the Editor:

**Figure:**
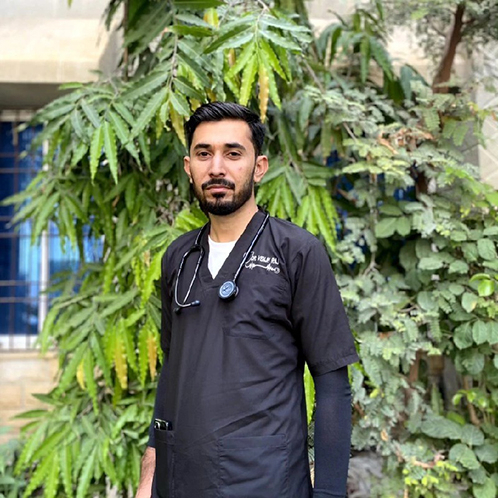

We recently read the article by Miura and colleagues.^1^ The authors' job of providing readers with this kind of in-depth insight was excellent. I appreciate the writers' efforts in conducting this research. Despite this, I believe that a few additions merit further thought and could strengthen the validity of this study.

First, after distal aortic excision, chronic dissection poses considerable difficulty and is generally associated with a high risk of intraoperative and postoperative complications. There is disagreement regarding when to intervene following the onset and complications of dissection. It can be challenging to directly compare different treatment techniques because patients assigned to medical treatment, thoracic endovascular aortic repair (TEVAR), or open surgery frequently have baseline comorbidities and disease severities that differ greatly. Older age, pulmonary illness, cerebrovascular disease, and aneurysm rupture were independent predictors of unfavorable outcomes; extent IV thoracoabdominal aortic aneurysm repair was linked to a decreased chance of unfavorable outcomes.^2^

Second, Even with improved surgical methods and neuroprotective techniques, 5% to 15% of postoperative neurological problems result from open repair. There have been fewer reports of spinal and brain problems with TEVAR than with surgery alone. Another end-organ perfusion strategy employed by certain centers involves combining endovascular flap fenestration with branch artery stenting in patients who present with malperfusion, and the reported incidence of neurologic sequelae from this approach is comparable to that following total endovascular resection. However, fenestration is not a treatment option for dilatation or rupture.^3^ Third, for the treatment of chronic difficult Stanford type B aortic dissection, there has been increased interest in TEVAR because of its lower early mortality and morbidity compared with open surgery.

However, there is still debate over the best course of action for patients with DeBakey IIIb dissections involving the thoracic and abdominal aorta, particularly those whose results from TEVAR for chronic Stanford type B aortic dissection have been inconsistent. With excellent 30-day and long-term outcomes, the current trial supports TEVAR as a successful therapy for chronic DeBakey IIIb patients with adequate anatomy, meaning that the patient has an isolated descending aneurysm rather than a thoracoabdominal aneurysm. Of the few patients without apparent regression, an anatomic reason was found. Almost all patients with imaging follow-up longer than 6 months showed significant aneurysm sac regression (≥1 cm) in the intervened-on segment.^4^

Lastly, Although the perioperative and intermediate-term outcomes of endovascular repair of chronic distal aortic dissection are favorable, a large number of patients have illness that is restricted to the thoracic aorta. Only 75% to 83% of treated segments experience false lumen thrombosis. Within a 3-year follow-up period, 37% of patients need endovascular or open surgical reintervention. For individuals with limited dissections or a high risk of open surgery, endovascular treatment is advised. Nevertheless, poor results are seen in patients with later stages of aneurysmal degeneration and extensive dissection.^5^

## Conflict of Interest Statement

The authors reported no conflicts of interest.

The *Journal* policy requires editors and reviewers to disclose conflicts of interest and to decline handling or reviewing manuscripts for which they may have a conflict of interest. The editors and reviewers of this article have no conflicts of interest.
